# Student learning outcomes, perceptions and beliefs in the context of strengthening research integration into the first year of medical school

**DOI:** 10.1007/s10459-017-9803-0

**Published:** 2017-11-11

**Authors:** Mayke W. C. Vereijken, Roeland M. van der Rijst, Jan H. van Driel, Friedo W. Dekker

**Affiliations:** 1 ICLON Graduate School of Teaching, Leiden University, P.O. Box 905, 2300 AX Leiden, The Netherlands; 2 Graduate School of Education, University of Melbourne, 234 Queensberry Street, Melbourne, VIC 3010 Australia; 3 Department of Clinical Epidemiology, Leiden University Medical Centre, P.O. Box 9600, 2300 RC Leiden, The Netherlands

**Keywords:** Undergraduate education, Research-teaching nexus, Student learning outcomes, Undergraduate research

## Abstract

Research integrated into undergraduate education is important in order for medical students to understand and value research for later clinical practice. Therefore, attempts are being made to strengthen the integration of research into teaching from the first year onwards. First-year students may interpret attempts made to strengthen research integration differently than intended by teachers. This might be explained by student beliefs about learning and research as well as student perceptions of the learning environment. In general, student perceptions of the learning environment play a pivotal role in fostering student learning outcomes. This study aims to determine whether a curriculum change intended to promote research integration fosters student learning outcomes and student perceptions of research integrated into teaching. To serve this purpose, three subsequent cohorts of first-year students were compared, one before and two after a curriculum change. Learning outcomes of these students were measured using scores on a national progress test of 921 students and assessments of a sample of 100 research reports of a first-year student research project. 746 Students filled out the Student Perceptions of Research Integration Questionnaire. The findings suggest that learning outcomes of these students, that is, scores on research related test items of the progress test and the quality of research reports, were better than those of students before the curriculum change.

## Introduction

The promotion of undergraduate students’ understanding of research is an important aim of medical education internationally (AAMC 1998; CanMeds 2015; GMC 2015). It puts emphasis on strengthening the integration of research into teaching in undergraduate medical education, for example, through curriculum interventions to promote students’ understanding of research (Mullan et al. 2014; Pruskil et al. 2009). Medical students find research integrated into their education stimulating for their learning process (Murdoch-Eaton et al. 2010), although students might be less enthusiastic about strengthening research integration by doing their own research projects. Previous studies have emphasized students concerns about research endeavors which could delay completion of their medical education (Funston et al. 2016; Siemens et al. 2010). Medical teachers are therefore challenged to explicate research in all their teaching in order for students to understand and value research for routine clinical practice, not just for physician-scientists (Laidlaw et al. 2012; Ribeiro et al. 2015). The aim of this study is to determine effects of strengthening research integration into teaching on student learning outcomes and student perceptions of research within undergraduate education in large cohorts of students. The term ‘research integration’ is used for all learning activities in which doing research or student engagement inresearch products and processes are an essential part of first-year undergraduate courses in the medical domain (cf. Healey and Jenkins 2009).

Several studies have placed importance on strong research integration for student learning. Research integration, for example in student research projects, traditionally takes place towards the end of the undergraduate medical curriculum (de Oliveira et al. 2011; Oliveira et al. 2013; Siemens et al. 2010). Especially for first-year students it may be difficult to experience aspects of research in courses within undergraduate education (Burgoyne et al. 2010; Oliveira et al. 2013). Teachers may feel that first-year undergraduates in higher education are not yet ‘open’ to research (Zamorski 2002). Furthermore, first-year students see themselves rather as an audience of research than involved in knowledge production (Jenkins et al. 1998). First-year students have positive expectations about doing research later in their degree (Smith and Rust 2007). However, students also report disadvantages of research integrated into teaching, such as staff overcoming their own challenges in dealing with teaching and research responsibilities (Healey et al. 2010). Thus, first-year students may interpret efforts made by teachers to explicate research differently from what was intended (e.g. van der Rijst et al. 2013). The present study therefore compares cohorts of first-year students when research is more prominently incorporated into courses using student perceptions of research integration and student learning outcomes as concepts.

**Fig. 1:**
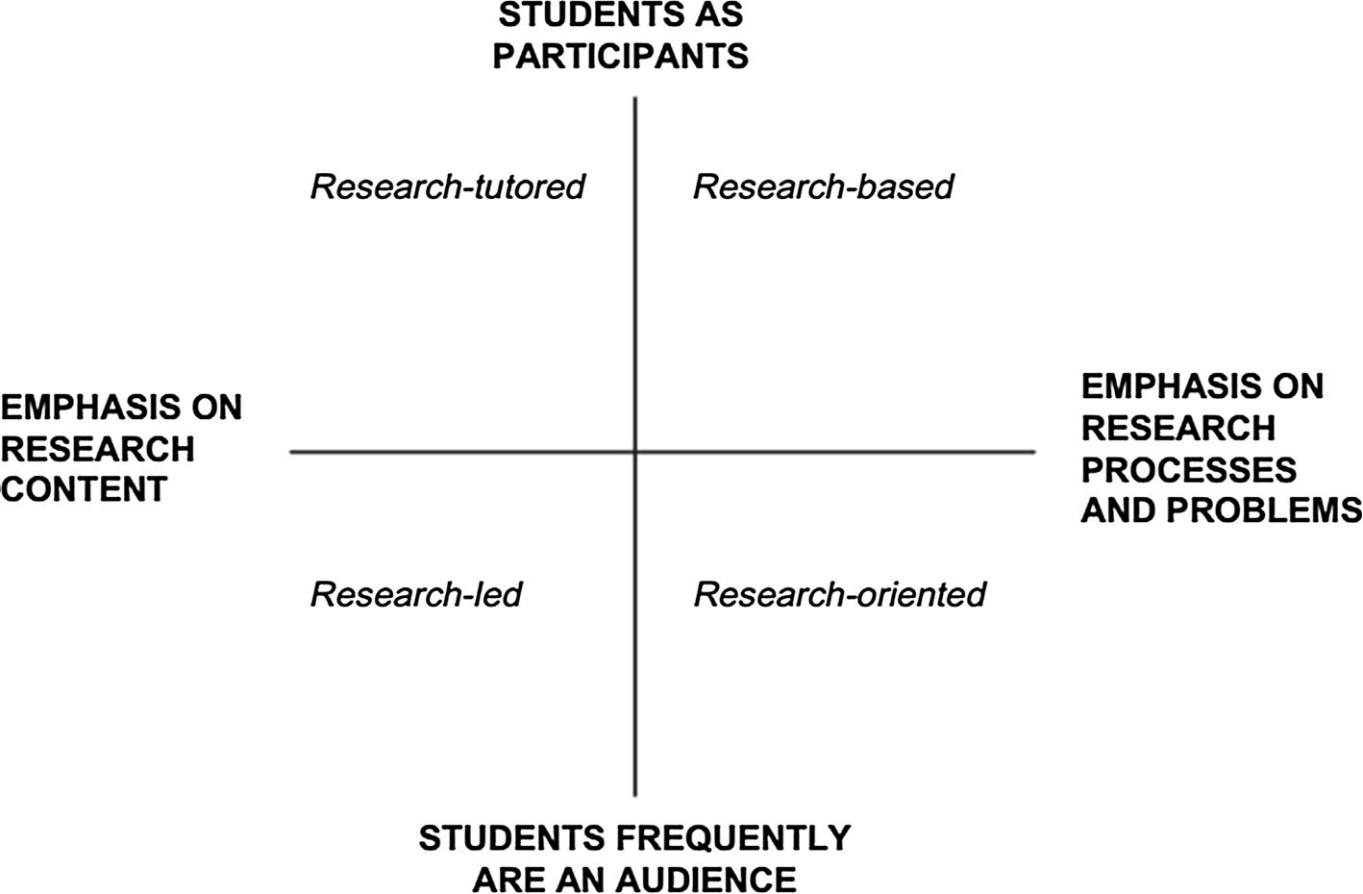
Two dimensions to describe research integrated into undergraduate courses (Healey and Jenkins 2009)

Research integrated into undergraduate courses can take different forms based on two dimensions (Healey and Jenkins 2009). The first dimension concerns the focus of the research elements that are integrated into courses and runs from research processes (e.g. data collection and analysis in regular courses) to research content (e.g. focus on student understanding of research findings through coursework). The second dimension describes the extent to which students are actively engaged in research through their courses and goes from students involved as an audience of research to students involved as participants in research in the sense that students engage in research activities during their courses. These dimensions create basically four ways in which it is intended to integrate research into courses (see Fig. 1).

It has been argued that these ways to integrate research complement each other in order to promote student perceptions of research and perceived student learning outcomes (Healey and Jenkins 2009).

### Relationships between student learning outcomes, beliefs and perceptions

Constructivist models for student learning in higher education from the field of educational psychology show that student perceptions of the learning environment play a pivotal role in promoting their learning outcomes (Biggs 1985; Prosser and Trigwell 1999). Student perceptions can provide a valid and reliable image of the learning environment, since students have extensive experience in making observations during their school careers (Marsh and Roche 1997; Spooren et al. 2013). Positive student perceptions directly influence specific learning outcomes like academic achievement, skill performance and motivation for learning (Lizzio et al. 2002). These models for student learning suggest that the relationships between learning outcomes and student perceptions of teaching are reciprocal. Thus student perceptions of the effectiveness of teaching facilitate effective learning and the other way around (Ramsden 1991), even in the first undergraduate year (Prosser and Trigwell 2014).

Student perceptions of the learning environment are related to student beliefs about learning. Beliefs are generally referred to as a set of (partly implicit) suppositions, or as a lens through which students interpret the world, which is relatively stable over time and courses (Pajares 1992). In addition to student beliefs, elements in the learning environment and prior learning experiences influence student perceptions of the learning environment as well (e.g. Ashwin and Trigwell 2012). In the present study we are primarily interested in student learning outcomes and student perceptions of research. However, in our study student beliefs are taken into account in order to interpret our results sensibly. In particular to explain student learning outcomes and perceptions by changes in the learning environment.

Undergraduate medical students in their penultimate year might hold a belief that research is of limited value to their learning process in clinical rotations, although their perceptions of research could change after participation in a student research project (cf. Murdoch-Eaton et al. 2010). Findings from a recent review study suggest that students, after a research experience, value research for their future career path (Chang and Ramnanan 2015). In terms of learning outcomes differences were found among students’ interpretations of what research entails and the perceived skills involved in research (Bierer et al. 2015; Murdoch-Eaton et al. 2010). Medical undergraduate students’ interpretations of research may be focused on hypothesis testing, knowledge production, data collection and discovering new things (Burgoyne et al. 2010). In addition to previous studies, this study focusses on conceptually related variables (i.e., student learning outcomes, beliefs about the value of research for learning and student perceptions of research) in a context of strengthening research integration from the first-year onwards.

Two research questions are addressed in this study. First, does research integrated into the first-year curriculum promote student learning outcomes within the domain of research? Second, do first-year undergraduate students perceive a stronger research integration, in a curriculum that aims to strengthen research integration?

## Educational context

Our study was conducted at the a University Medical Center (UMC) in the Netherlands. Staff members at the UMC have responsibilities in patient care, research and teaching. The medical undergraduate program was structured in a two cycle model (Patrício and Harden 2010). A weighted lottery procedure based on students’ grade point average (GPA) in secondary education was used for first-year student admission for all cohorts in this study. Students with a high GPA are more likely to be admitted. Every academic year 330 students, usually 19 years old, start studying medicine in the UMC.

**Fig. 2:**
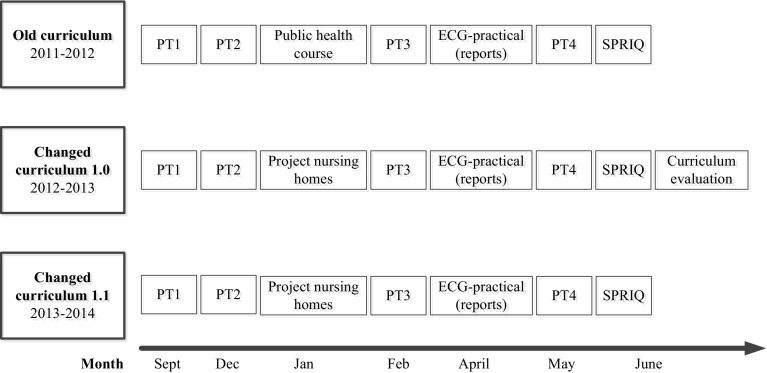
Timeline of the curriculum change including progress tests (PT), student research activities and Student Perception of Research Integration Questionnaire (SPRIQ)

A curriculum change was implemented in the first cycle from the 2012–2013 academic year. A timeline of the curriculum change is shown in Fig. 2. Before 2012–2013, the first year curriculum (baseline) was predominantly based on theoretical classes augmented by learning activities in small groups (here: old curriculum). The aim of the curriculum change was to strengthen the integration of research in undergraduate courses. The changes in the curriculum design were informed by the integration continuum with full integration at one end and discipline-based education at the other (Harden 2000). In this study, the old curriculum is defined as ‘harmonised’ in the sense that teachers consulted each other and communicated about their courses. The changed curriculum can be classified as ‘multi-disciplinary’, as clearly identified subjects were brought together in a single course with an integrated theme aiming to provide authentic learning experiences (Harden and Laidlaw 2012, p. 94). In the changed curriculum teachers from basic sciences and clinical disciplines were brought together to develop courses collaboratively. The duration of courses was between two and five weeks and courses were developed within separate disciplines. Student assessment took place at the end of a course mainly by multiple-choice question examination. After the 2012–2013 academic year the changed curriculum (version 1.0) was evaluated with students and teachers. As a result, minimal adaptations were made in order to improve student learning experiences, for instance to improve the spread of study load (version 1.1). The old and changed curricula were developed according to the Dutch Blueprint (NFU 2009). This study was designed to make a comparison between student learning outcomes within a curriculum using strategies to foster harmonisation and a curriculum aiming to promote multi-disciplinary strategies in order to strengthen research integration.

### Fostering research integration

Regarding research integration, the curriculum change aimed to promote authenticity of student learning experiences. To this end, epidemiology teachers have collaborated with primary care teachers in developing a first-year student research project. In particular, a classical three-week course on public health, epidemiology and biostatistics in the old curriculum was replaced by a small student research project for all students (cf. Dekker et al. 2009). Students collect data about co-morbidity, medication, care dependency and cognition among three patients during an early clinical experience in nursing homes directly at the start of medical education in September. Students enter their data into an online database in order to establish a large dataset (300 × 3 = 900 patients). In December the students return to the nursing homes for one day to repeat their data collection and to come up with their individual research questions at the ‘bedside’. In the new two-week course thereafter basic knowledge and skills were taught to enable them to answer their own research question (see Fig. 2). In two small group sessions students have practiced formulating a research question and have learned to understand the structure of a research paper. Students had a few lectures on epidemiology and basic statistics and practice in simple data analysis. Then students have spent two days to analyze their data and to answer their own research question. They have written a two-page research report and present their findings to their peers in a small group session. All students were actively involved as participants in research doing their own research project as a learning activity (cf. Healey and Jenkins 2009).

In both curricula students also participate in a practical in April in which they collect electrocardiographs (ECGs) of their peers, they formulate a research question, analyze the data and present findings. Emphasis was on promoting student understanding of study designs, statistics and written and oral presentation of findings. Students had written a short research report in a small group session. The ECG-project was already developed to incorporate research more explicitly and so it was maintained with minimal adaptations. Student instruction in this course was extended by one small group session involving peer feedback on academic writing.

Besides this all teachers were encouraged by a curriculum committee to explicate links between research and clinical practice within their courses where possible (e.g. Laidlaw et al. 2012). To that end, curriculum developers discussed the student research projects with all teachers. These discussions compelled teachers to explicate their ideas for strengthening research integration appropriate to their field and course.

## Methods

### Data collection and instruments

Cognitive learning goals were tested four times a year using a national progress test (PT) (Muijtjens et al. 2008). In the Netherlands, staff members of five universities take part in writing test items covering knowledge across all disciplines and domains relevant for the medical degree. The first PT took place in September, the second PT in December, the third PT in February and the final PT in May (see Fig. 2). The aim of the PT is to determine the growth of individual student knowledge longitudinally and the PT contributes to more reliable and valid decision making for future competence or retention of knowledge (e.g. Schuwirth and van der Vleuten 2012). Student scores of the third (March) and fourth (May) PT in the first year were collected. Nine of the 200 items in total per PT reflected student knowledge about scientific research and methods and was assessed in closed format (‘true’; ‘false’; ‘do not know’). The ‘do not know’ option, scored as a neutral 0, is preferred over negative marking in the PT, since this option allows students to avoid guessing without penalty (McHarg et al. 2005; Muijtjens et al. 1999). Students scored + 1 point for every correct answer, − 1 for an incorrect answer and 0 points when they answered ‘do not know’. Scores on the PTs were converted to a scale from 0 to 100 for further analysis.

In the ECG-practical students wrote an extended abstract as a research report. The reports were rated using a rubric developed for the purpose of this study. The raters were trained during the development process of the rubric to enable informed decisions about criteria and descriptors adequately capturing key aspects of student performance (e.g. Cook and Hatala 2016). Two batches of 50 reports were randomly selected (old and changed curriculum) and all were assessed blindly and anonymously by six trained raters (an educationalist, epidemiologist, pediatrician, physiologist and two-third-year students) on a grading rubric designed for this study. The rubrics contained 11 criteria and three descriptors (range 0–22) regarding (1) consistency across introduction, method, results and discussion and (2) structural characteristics of the text in order to assess written presentation of student research findings (see "Appendix"). The intraclass correlation coefficient (ICC) for the average measure using absolute agreement with six fixed raters was .81, suggesting a good interrater reliability (Streiner and Norman 1995). We used the average measure because our raters were a random sample of all possible raters and the reports were selected randomly as well (Shrout and Fleiss 1979).

### Student beliefs and perceptions

To measure student perceptions of research integration and student beliefs about research we administered the Student Perception of Research Integration Questionnaire (SPRIQ) (Visser-Wijnveen et al. 2016). The scales include: (1) critical reflection on how research results are produced; (2) student participation as a researcher in learning activities; (3) familiarity with current research done by staff; (4) interest and motivation for research; (5) beliefs about the value of research for their learning; and (6) perceived quality of the learning environment. We slightly adjusted general item wordings such as changing ‘scientific domain’ to ‘medicine’ in order to fit the medical context (Vereijken et al. 2016). We added a scale about beliefs about the value of research for clinical practice. All 30 items were answered on a 5-point Likert-scale. The ‘quality’ scale was included because students’ opinions on the general quality of teaching during the first academic year could influence their scores on the other scales. Table 1 shows the scales, reliability and sample items of the version of SPRIQ that was used.

**Table 1:** Scales, reliability and sample items of the Student Perception of Research Integration Questionnaire

Scales	N items	Sample items during this academic year…	α^a^
*First*-*year student perceptions*			
Critical reflection on research	4	… attention was paid to research methods	.63–.75
Participation in research	5	… as a student I felt involved in research	.82–.85
Familiarity with current research	5	… I became familiar with the research carried out by my teachers	.72–.79
Motivation for research	4	… I became enthusiastic about research in medicine	.81–.83
*Other*			
Beliefs about the value of research for practice	6	Scientific skills are important for being a doctor	.84–.88
Beliefs about the value of research for learning	3	… my learning is stimulated when education is grounded in research	.80–.85
Quality of learning environment	3	… the teachers carried out their instruction adequately	.69–.75

^a^Cronbach’s alpha varied slightly per year of data collection; lowest and highest are reported indicating acceptable to strong internal consistency of scales (Cohen 1998)

### Participants

All first-year students who started their studies in the old or changed curriculum 1.0 and 1.1 were invited to participate in this cohort study. We included two groups of students within the changed curriculum to be able to check for cohort effects. Data were collected during lectures from May to June of every academic year (see Fig. 2). We distributed the hardcopy questionnaires to all attending students, who were asked to fill out the questionnaire for all courses taken up till then. They were asked for permission for their unique student identification number to be used, so that we could send the questionnaire to the students not present at the lecture. A reminder was sent by e-mail to those students who did not respond to the first invitation. Ethical approval was granted by the UMC Research Ethics Committee.

### Analysis

#### Progress tests

A mean score for items about scientific research and methods in PT1 and PT2 before the student research project in the nursing homes was calculated per curriculum, and also for PT3 and PT4 after this project. We compared the mean scores on the items using independent t-tests (changed curriculum 1.0-old curriculum; changed curriculum 1.1-old curriculum). In addition, we used linear regression to adjust for the mean score of items about scientific research and methods in PT1 and PT2 before the student research project. In a separate linear regression analysis, we adjusted for the mean overall score on PT3 and PT4.

#### Research reports

A mean score per report, over all reports and raters was calculated. Thereafter reports were decoded, indicating whether a report was written in the old or changed curriculum. Then reports were divided based on the two curricula. After that we compared the scores per curriculum, thus on all the raters and reports using an independent t-test.

#### SPRIQ

Means for every scale of the SPRIQ were calculated for all cohorts. After that, scale means per curriculum were compared using independent t-tests (changed curriculum 1.0-old curriculum; changed curriculum 1.1-old curriculum). A confidence interval of 95% was applied for all t-tests.

## Results

### Student learning outcomes

Student scores of the research related items of PT1 and PT2 were lower in the changed curriculum in 2012 (mean difference − 5.39 (95% CI [− 7.20; − 3.60]). The mean scores of students on research related items of PT1 and PT2 were higher in the changed curriculum in 2013 (mean difference 4.26 (95% CI [2.33; 6.19])). The mean score of the research-related items of PT3 and PT4 in the changed curriculum in 2012 was significantly higher compared to the old curriculum (Table 2). After correction for the corresponding mean score of research-related items of PT1 and PT2 the adjusted difference was 14.73 (95% CI [12.29, 17.17]). When controlling for student mean scores on all items of PT3 and PT4 the difference between the old and changed curriculum 1.0 was 9.62 (95% CI [7.45, 11.78]). In the changed curriculum 1.1the mean score on the research-related items of PT3 and PT4 was also significantly higher compared to the old curriculum (Table 2). This difference remained after controlling for student scores on research-related items at PT1 and PT2 (adjusted difference 15.98; 95% CI [13.48, 18.48]). After controlling for student scores on all items of PT3 and PT4 the effects were not materially different (adjusted difference 14.55; 95% CI [12.31, 16.77]). With regard to the student research reports, a significant difference was found between the old and the changed curriculum 1.1 in favor of the changed curriculum (difference 5.90; 95% CI [4.89, 6.91].

**Table 2:** Mean scores student learning outcomes and scale means on the Student Perception of Research Integration Questionnaire per cohort (5-point Likert scale) before and after the curriculum change

Scales	Old curriculum Mean (SD)	Changed curriculum 1.0 Mean (SD)	Changed curriculum 1.1 Mean (SD)
*Student perceptions*			
Critical reflection	2.98 (.66)	3.24 (.61)^a^	3.44 (.63)^a^
Participation in research	1.94 (.69)	2.20 (.72)^a^	2.44 (.71)^a^
Familiarity with current research	2.65 (.68)	3.02 (.72)^a^	3.09 (.62)^a^
Motivation for research	2.71 (.78)	2.97 (.81)^a^	3.11 (.77)^a^
*Other*			
Beliefs on value of research for practice	3.64 (.67)	3.56 (.76)	3.75 (.52)
Beliefs on value of research for learning	2.99 (.81)	2.96 (.84)	3.21 (.77)^a^
Quality learning environment	3.80 (.51)	3.76 (.61)	3.75 (.52)
*Student learning outcomes*			
Student research reports	8.93 (2.77)	No data	14.83 (2.31)^a^
Research related progress test (PT) items (PT1 and PT2)	14.25 (12.32)	8.85 (11.78)	18.51 (13.10)^a^
Research related progress test (PT) items (PT3 and PT4)	16.47 (14.26)	28.93 (16.21)^a^	34.41 (17.29)^a^

^a^Indicates this scale mean is higher than in the old curriculum (t-test; *p* ≤ .05)

### Student beliefs and perceptions

In total 746 first-year students filled out SPRIQ (response rate 75.4%). A vast majority of the respondents had started studying medicine as their first degree (n = 692). Table 3 provides an overview of the data collection periods and characteristics of the respondent group. The majority of the participating students was female, which indicates that the sample is representative for the medical student population (e.g. van der Velden et al. 2008).

**Table 3:** Characteristics of data collection and cohorts of first-year students

Curriculum	Data collection	N_respondents_	Female	Response rate (%)	Average age (years)
Old	May/june 2012	261	187 (71.6%)	85.9	19.7
Changed 1.0	May/june 2013	248	147 (59.3%)	75.2	19.4
Changed 1.1	May/june 2014	237	149 (70.6%)	62.2	19.5

Table 2 shows the scale means on SPRIQ for the previous curriculum in comparison to two groups in the changed curriculum. Abbreviations indicate the scale names. Scores on the perception scales ‘critical reflection’, ‘participation’, ‘familiarity’ and ‘motivation’ are significantly higher in the changed curriculum 1.0 and 1.1 in comparison to the old curriculum. Scale means on perception scales are the highest for the changed curriculum 1.1. With regard to beliefs about the value of research for future practice and the perceived quality of the learning environment, no differences were found between curricula. In the changed curriculum 1.1 students held a significantly stronger belief about the value of research for their learning than in the earlier curricula. ‘Critical reflection on research’ was experienced the most, then ‘familiarity with current research’ and ‘motivation for research’ in all three groups. Perception scores on ‘participation in research’ were the lowest of four scales in both curricula, although students felt significantly more involved as participants in research through the learning activities in the changed curriculum.

## Discussion

The findings of this study suggest that strengthening research integration had a positive effect on research related first-year student learning outcomes. Particularly on research related items of a national progress test and research reports from a student research project. The results indicate that first-year medical students recognized a stronger emphasis on research within courses after a curriculum change that was intended to promote student engagement in research. The first-year students tended to believe that research is important for their future careers in clinical practice. In sum, the findings suggest that the curriculum seemed to improve students’ perceptions of research integration, yet seemed not to affect their beliefs about the value of research.

The curriculum change described in this study consisted of interventions with regard to assessment, collaboration between disciplines in teaching and duration of courses in order for students to benefit from an emphasis on strengthening the integration of research and teaching. Since the study design was observational in nature, causal conclusions between the curriculum change and student learning outcomes should be drawn with caution. Nevertheless, this study attempted to answer the call made in comparative curriculum studies to use the best possible comparison group (cf. Pruskil et al. 2009). The data used in this study reflect first-year student learning outcomes and student perceptions of research integration. In higher education research in general it is argued that the quality of student learning outcomes depend on factors related to the quality of student learning as a process, such as students’ prior learning experiences, student perceptions of the learning environment and their approaches to learning (e.g. Prosser and Trigwell 2014). Approaches to learning indicate whether students focus on, for example, transmission, reproduction or production of knowledge (Prosser and Trigwell 2014). The present study, therefore, contributes to the quality of student learning in medical education, improving students’ research knowledge through learning activities within the undergraduate program (e.g. Laursen 2015). The findings of this study are based on high response rates, validated questionnaires and two types of learning outcomes. Most importantly, our findings can be explained by conceptual relationships between student learning outcomes, student beliefs about the value of research for learning and student perceptions of research integrated into courses (Pajares 1992; Prosser and Trigwell 2014).

Students performed better on research-related items in a national progress test and on written student research reports after the curriculum change. An explanation for this is that the students in the changed curriculum were actively engaged in an authentic student research project before writing the reports and doing the progress tests. In the learning process in general student learning outcomes are influenced by factors such as student perceptions of teaching, student motivation and values (Biggs 1985; Prosser and Trigwell 1999). In that sense the learning outcomes measured in this study were closely related to the learning process whereas previous studies into research integration and medical student learning could be further away from the student learning process. In a recent systematic literature review, Chang and Ramnanan (2015) suggest that previous attempts made to improve student learning and research-related outcomes were mainly informed by student perceptions of research and long-term research outcomes such as presentations at conferences and peer-reviewed publications. This might raise questions about variables used in medical education research into research integration, such as research output, to inform curriculum decision making and to improve the quality of student learning.

Teachers may feel that first-year students might not yet be open to research (Zamorski 2002), which could be the case for undergraduate medical students in general (Burgoyne et al. 2010; Murdoch-Eaton et al. 2010). Our findings suggest that students do recognise research integration and, more importantly, that a curriculum change including a first-year student research project can promote student perceptions of research in the first undergraduate year of medical education. Students recognise research in courses in several ways according to the scales used in the SPRIQ. The results show that, although student perceptions of research increased on all scales after the curriculum change, participation in research was experienced the least and critical reflection on research the most. This indicates that the perceived ways in which research is actively included in student learning are complementary. Teachers therefore should be encouraged to use a range of modes in order to actively include research even in first-year student education.

Small differences were found between student beliefs about research before and after the curriculum change. The relatively stable nature of beliefs can provide an explanation for this (Pajares 1992). Students already tend to believe that research is important for physicians’ practice when they enter medical education. Despite the nature of beliefs, this indicates that the differences found in learning outcomes and student perceptions in our study can be explained by changes in the learning environment (e.g. Ashwin and Trigwell 2012).

Future studies are needed to provide insight into student learning processes in courses or projects in which research is strongly integrated in order to improve the quality of student learning about research. Future studies in medical education research might benefit from careful consideration of variables and designs used to foster high quality learning outcomes in medical education research into research integration. For example, by focusing on relations between student perceptions of research in teaching, the way students approach learning (i.e., knowledge transmission, reproduction, production) and student learning outcomes (e.g. Prosser and Trigwell 2014; van der Rijst 2017).

## Conclusions

This study was conducted to improve our understanding of the relation between student learning outcomes, beliefs about the value of research for student learning and student perceptions of research integrated into courses by investigating first-year student learning in the context of a curriculum change. First-year students performed better on research related learning outcomes in a national progress test as well as in writing research reports in a local student research project. Students in a changed curriculum, intended to strengthen research integration, recognized a stronger emphasis on (1) critical reflection on research, (2) participation in research activities, (3) familiarity with research done by the staff and (4) being motivated for research in medical education. Students tended to have a strong belief in the value of research for their future clinical practice. Implications of this study inform curriculum decisions about integrating research into courses using multi-disciplinary strategies to foster research integration (cf. Harden and Laidlaw 2012). In sum, strengthening research integration in undergraduate courses is feasible in a limited amount of curriculum time, and can lead to enhanced student perceptions and associated learning outcomes. The findings indicate that student beliefs about the value of research are less fluent in comparison to student perceptions of research and learning outcomes in the domain of research. This study contributes to an emerging body of knowledge about improving students’ research knowledge through student engagement in research as a pedagogy i.e., through learning activities within the undergraduate curriculum.

## Appendix: Grading rubric first-year student research reports (translated from Dutch)

**Table:**

Score per criterion	0	1	2
*Consistency*			
Introduction	Research question (RQ) is missing; no indication of relevance, no rationale	Lack of argument(s) underpinning the RQ	Introduction provides clear arguments underpinning RQ, aim or hypothesis
Method	Unbalanced in terms of size; either overlong or lacks key information about participants and analysis	Analysis suits the RQ. Mainly replicable, lacks detail	Clear to the reader. Enables replicability appropriate to a short report
Results	Contains redundant information, students’ interpretations or opinions	Factual display of results. Either too limited or too detailed	Comprehensive and factual display of results
Discussion	No indication of a limitation, conclusion does not fit RQ and results	Appropriate conclusion and a limitation. Either overgeneralized implications or lacks explanation of results (previous studies)	Results are related to previous or future research. Contains limitations, implications, main conclusion and answer to the RQ
*Structural characteristics*			
Title	Does not reflect the message, raises different expectations	Partly reflects the main message	Covers the main message
Structure of the text	No order (introduction—method—results—discussion)	In logical order, at times repetition or overlap	Coherent, to the point, reads easily
Language (terminology)	Style and spelling errors, inconsistent use of scientific language	Nearly flawless and consistent use of scientific language	No errors, consistent use of scientific language
Comprehensiveness	Text is not confined to key issues. (Abbreviations like MET and QRS are common language)	Key issues are clear; missing are details needed to answer the RQ	Key issues are clear; contains relevant information in order to answer the RQ
References	No references	Some information is missing or not in Vancouver-style	Full reference list in Vancouver-style
Tables and figures	Messy, too large or too small on the page. Overlaps text	Make an orderly impression. Lay-out does not fully support the text	Numbered tables, support the message in the text
Attractiveness abstract	Does not encourage further reading	Raises the reader’s interest	Report fosters further reading. Can not wait to read more
Total score report			
